# “Hopeless but supported in that hopelessness”: a qualitative study of how people experience talking to a GP about suicide

**DOI:** 10.3389/fpsyt.2026.1744949

**Published:** 2026-02-11

**Authors:** Sophia Fedorowicz, Robert C. Dempsey, Naomi Ellis, Christopher Gidlow

**Affiliations:** 1 Centre for Health and Development, Staffordshire University, Stoke-on-Trent, United Kingdom; 2 Department of Psychology, Faculty of Health and Education, Manchester Metropolitan University, Manchester, United Kingdom; 3 School of Medicine, University Road, Keele University, Staffordshire, United Kingdom; 4 Midlands Partnership University NHS Foundation Trust, Research and innovation Department, St Georges Hospital, Stafford, United Kingdom

**Keywords:** lived experience, mental health, primary care, qualitative, suicide

## Abstract

**Introduction:**

There is evidence that help-seeking escalates in the weeks before death by suicide, with general practice being the most common last point of contact. The experiences of people seeking support for suicidal thoughts and feelings in primary care is under-explored. Understanding the perspectives of people experiencing suicidal thoughts and feelings may identify innovative ways to assess risk in primary care in a safe and collaborative way, allowing more opportunity for intervention. The aim of the current qualitative study was to explore individual experiences of talking to a GP about suicide to understand how they perceive these interactions.

**Methods:**

This study was developed with people who have lived experience of suicidality and support seeking, who also supported the interpretation of data and informed the dissemination plan. A participatory, consultative approach was adopted, with experts by experience involved flexibly at multiple stages of the research. Participation was strengths-based and care-informed, prioritising choice, comfort, and psychological safety. Data were collected using an online qualitative survey that was distributed using social media. Forty-one responses were inductively analysed using Reflexive Thematic Analysis. Participants were aged between 19 and 67 years old, 29 were female, nine male, two non-binary, and one did not disclose their gender.

**Results:**

Three overarching themes were identified: 1) Challenges disclosing suicidal thoughts and feelings: “*I wish she would just say suicide*”; 2) GP limitations: “*I felt my medical needs were met, but not necessarily my mental health needs*”; 3) Creating a safe space: “*He made it normal, not embarrassing or weird*.” This study identified a range of factors influencing how people experience talking to a GP about suicide, including stigma, fear of the consequences of disclosing suicidality, the resources available to GPs generally (e.g., training and knowledge of suicide prevention), and GPs’ active listening skills.

**Discussion:**

These findings have implications for practice largely connected to a need for relationally informed responses to suicidality that promote more compassionate, contextually responsive mental health care. Methodologically, the paper demonstrates the value of participatory, lived experience–led research grounded in trust, reciprocity, and collaboration beyond tokenism.

## 1 Introduction

Suicide is a serious, complex, and preventable public health problem that has devastating consequences for individuals, families, and communities (1). Globally, approximately 700,000 people die by suicide each year and many more attempt suicide (1). In the United Kingdom, 7,055 deaths by suicide were registered in 2023 (2). The rate of suicide in England in 2023 were the highest since 1999, and both Scotland and Wales have seen increases (2). Reducing suicide deaths remains a priority for public health practitioners, health and social care professionals and the United Nations (sustainable development goal target 3.4.2.) (3).

Contact with primary care providers in the time leading up to suicide is common. A systematic review found contact with primary health care was highest in the year prior to suicide with an average contact rate of 80% (4). Luoma et al. (5) found three of four people who died by suicide had contact with primary care within the year of their death and 45% had contact with primary care within one month of suicide (5). There is evidence that help-seeking escalates in the weeks before death, with general practice being the most common last point of contact (6). Almost half of all people who died by suicide had their final consultation with their General Practitioner (GP) in the month before death and one-sixth in the week before death (7). Identifying those at highest risk for suicide during these primary care contacts is crucial as the evidence indicates that there is opportunity for intervention in the weeks and months before death.

Identifying those at risk of suicide is challenging. Several tools aim to support healthcare professionals to identify those at highest suicide risk such as the Beck Hopelessness Scale (8) and the SAD PERSONS scale (9). However, such measures generally list common risk factors including mental illness diagnosis, physical illness, negative thinking styles, life stressors and minority group status. The non-specific nature of these risk factors makes it very difficult for healthcare professionals to accurately identify those at high risk using this guidance alone because any member of the public could be experiencing several risk factors regardless of suicidality (10). Evidence indicates that the dichotomous yes/no response invited by the self-report format of suicide risk assessments can fail to identify individuals at risk, as some participants may omit or decline to answer (11). There is also evidence that healthcare professionals use unvalidated tools to assess for suicide risk in their practice, which raises further concerns around efficacy and consistency in assessing risk (12).

There is growing recognition of the role of social factors in suicidality related outcomes including the influence of peers and social norms (13) and socio-cultural factors such as nationality, ethnicity, and gender (14). Growing evidence highlights the profound impact of socio-economic factors on suicide risk, underscoring the need to move beyond individual-level explanations and consider broader social determinants. Two of the most robust predictors of suicide are indicators of material wealth, specifically unemployment and low socio-economic status (15). While social and economic factors, such as unemployment and socioeconomic status, are associated with suicidal experiences, individual prediction based on these factors is limited (10, 16). Evidence also indicates that national suicide rates tend to increase during periods of economic recession (17). Consequently, rather than centering suicide prevention efforts solely on individual-level interventions, there is an argument for broader public initiatives to enhance social and economic conditions (18). Recognising the importance of the social environment is essential for effective suicide prevention, yet dominant theories have largely neglected how structural inequality, colonisation, and intersecting systems of oppression, privilege, and power shape vulnerability to suicide, an omission with significant implications for prevention efforts (18).

In the UK, the National Institute for Health and Care Excellence (NICE) guidance explicitly advises against using suicide risk assessment tools and encourages reducing the reliance on risk stratification approaches and taking a holistic approach that considers a person’s safety and needs (19). Cole-King and Platt (20) identify the current approach to assessing suicide risk in people experiencing suicidal thoughts and feelings and responding only to those identified as “high risk” as fundamentally unsound practice which relies on unreliable stratification tools (20). Approximately 60% of GPs are unaware of published suicide prevention guidelines (including local, national, or international guidance) and there is marked variation in practice regarding how healthcare professionals conduct suicide risk assessment (12, 21).

There remains a lack of focus on the perspectives of people seeking support for suicidal thoughts and feelings in primary care, with a paucity of in-depth qualitative work exploring experiences of the risk assessment process. This is important to understand the complexity of suicidality and recognise that people are connected to their context (22). Considering the social context in which people experience suicidal thoughts and feelings can help to identify potential strategies that can buffer against these feelings (23). This involves creating space for alternative ways of thinking and being, including the meaningful inclusion of lived experience, the perspectives of marginalised communities, and other viewpoints often excluded by dominant institutionalised knowledge practices (24).

In this study, we use the term *suicidality* to refer to the spectrum of experiences related to thoughts, intentions, communications, planning, and behaviours associated with the desire to end one’s life. This usage is informed by O’Connor and Nock, who describe suicidal behaviour as encompassing both the psychological processes underlying suicidal thoughts and the actions individuals may take toward ending their lives, arising from a complex interplay of cognitive, emotional, and social factors (16).

The aim of the current qualitative study was to explore individual experiences of talking to a GP about suicide to understand how they perceive these interactions. This study was developed with people with lived experience of suicidality who also supported the interpretation of data and dissemination plan.

## 2 Methods

### 2.1 Design

This cross-sectional, descriptive-exploratory design allows access to personal narratives about talking to GPs about suicide. This study used a qualitative online survey design. Online qualitative surveys produce data that is assumed to be “thin”; however, these surveys can also generate rapid insights into how practice may affect people in varied ways (25). They offer an accessible and engaging way to gather rich insights from a diverse range of participants, particularly those who may be excluded from more traditional forms of qualitative research (25, 26). These surveys provide participants with a degree of autonomy and control, for example, allowing them to complete the survey at a time and place that suits them, and to withdraw easily if the survey does not interest them or meet expectations (25). The anonymous nature of participation can also be especially valuable for individuals from highly stigmatised groups (25). While qualitative surveys lack non-verbal cues, they allow participants to share their experiences anonymously and at their own pace, enabling participation from individuals who might not engage in interviews or focus groups, and providing access to perspectives that are often hard to reach, particularly from marginalised groups. Choosing the most appropriate method is central to rigorous qualitative research, and the involvement of people with lived experience in developing the survey was critical in ensuring the design was sensitive, accessible, and meaningful. This collaborative process aligns with a critical suicidology approach, foregrounding participants’ voices, attending to social and structural contexts, and emphasising reflexivity in both design and analysis.

This study draws on Critical Realism and Critical Suicide Studies, frameworks that more effectively situate suicide within broader contexts. Critical Realism stresses that there is a reality that exists independent of our thoughts about it (27). While observing participants’ experiences can enhance confidence in their reality, the knowledge produced is inherently constructed and influenced by the researcher’s perspectives and interpretations (27). Critical Suicide Studies engages in reflexive knowledge-making practices, critically examining its own assumptions and the potential for its approaches to inadvertently obscure forms of social exclusion, colonial violence, racism, patriarchy, consumerist, and transactional models of healthcare, and other systemic injustices (28). The participants' own language and meanings are prioritised, avoiding the imposition of pre-existing theoretical frameworks, to ensure that interpretation remains grounded in lived experience.

### 2.2 Lived experience involvement

**Figure 1:**
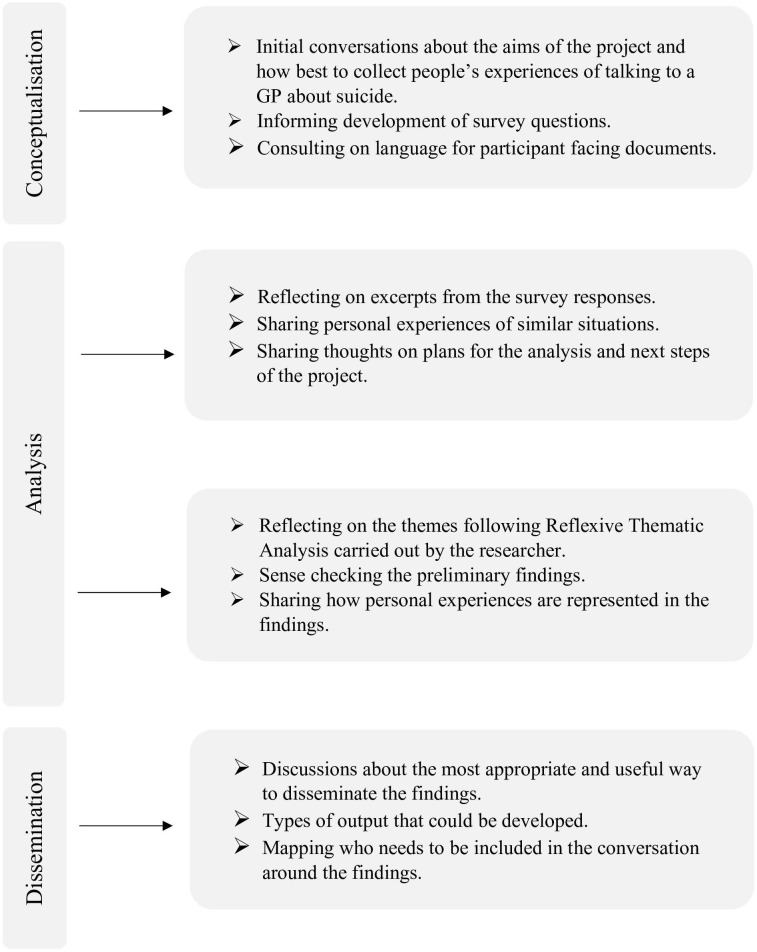
The lived experience involvement throughout the life cycle of the study.

The current study employs an approach that facilitates engagement with diverse meanings through attentive listening to first-person and lived experience accounts (**Figure 1**), thereby contributing valuable perspectives to broader suicidology (24). Prioritising lived experience, necessitates an ethical framework that moves the field beyond medically reductionist, technological, and ahistorical accounts of suicide toward a nuanced, contextually grounded, and historically and politically informed moral engagement (24).

Members of a lived experience-led organisation (Expert Citizens C.I.C.) who have personal lived experience of suicidality and support seeking volunteered to support the current project that aimed to explore individual experiences of talking to a GP about suicide. The current study was approved by University of Staffordshire Ethics Committee (approval number SU19-111). All participants gave informed consent prior to taking part in the study.

There is no “one-size-fits-all” approach to involving people with lived experience in research processes and therefore each study requires a tailored method (29). Caution must be employed as researchers and traditional research methods can replicate existing socio-economic inequalities and injustices through unfair and unjust structures and practices (30). The paucity of the perspective of people seeking support for suicidal thoughts and feelings in primary care in the literature can limit individual’s ability to make sense of their own experiences because they cannot access narratives that resemble their own (30). Involving people with lived experience in research as collaborators, as well as participants, contributes to dismantling these conditions of epistemic injustice by acknowledging people’s experiences as a credible source of knowledge and providing a space for those experiences to be known. In this current work, “lived experience” refers to the experiences of people who are members of a community impacted by a social issue, in this case experience of suicidality and support seeking (15). These experiences contribute to the development of specific knowledge and wisdom grounded in the insight gained from lived experience (31).

Expert Citizens C.I.C. are a non-profit organisation based in Stoke-on-Trent, England. The organisation is “built by and for people with lived experience”. It works with people with experiences of homelessness, drug and alcohol use, contact with the criminal justice system, domestic abuse, violence, exploitation, mental health challenges and many other types of social injustices to create positive changes in how they are supported by services and wider systems. Members of Expert Citizens volunteered to collaborate with SF on the project following a brief introduction to the topic and aim of the study. As the topic and direction of the work had already been determined owing to it being a part of a PhD, a “co-produced” study was not feasible, therefore it was felt that offering the volunteers a consultation role throughout the life cycle of the project was most appropriate. This role was flexible, allowing for individuals to drop in and out of a steering group for the project and collaborate on the activities that interested them, or that they felt comfortable with, rather than being required to attend every session. This “strengths-based” approach was informed by conversations with the Expert Citizens Volunteer and Network Co-ordinator, who had worked with the members for several months/years. This way of working was modelled on the “care-ful” research approach to suicide research (32) and built care into the way SF worked with the group of volunteers, prioritising physical and psychological safety.

### 2.3 Qualitative survey

**Figure 2:**
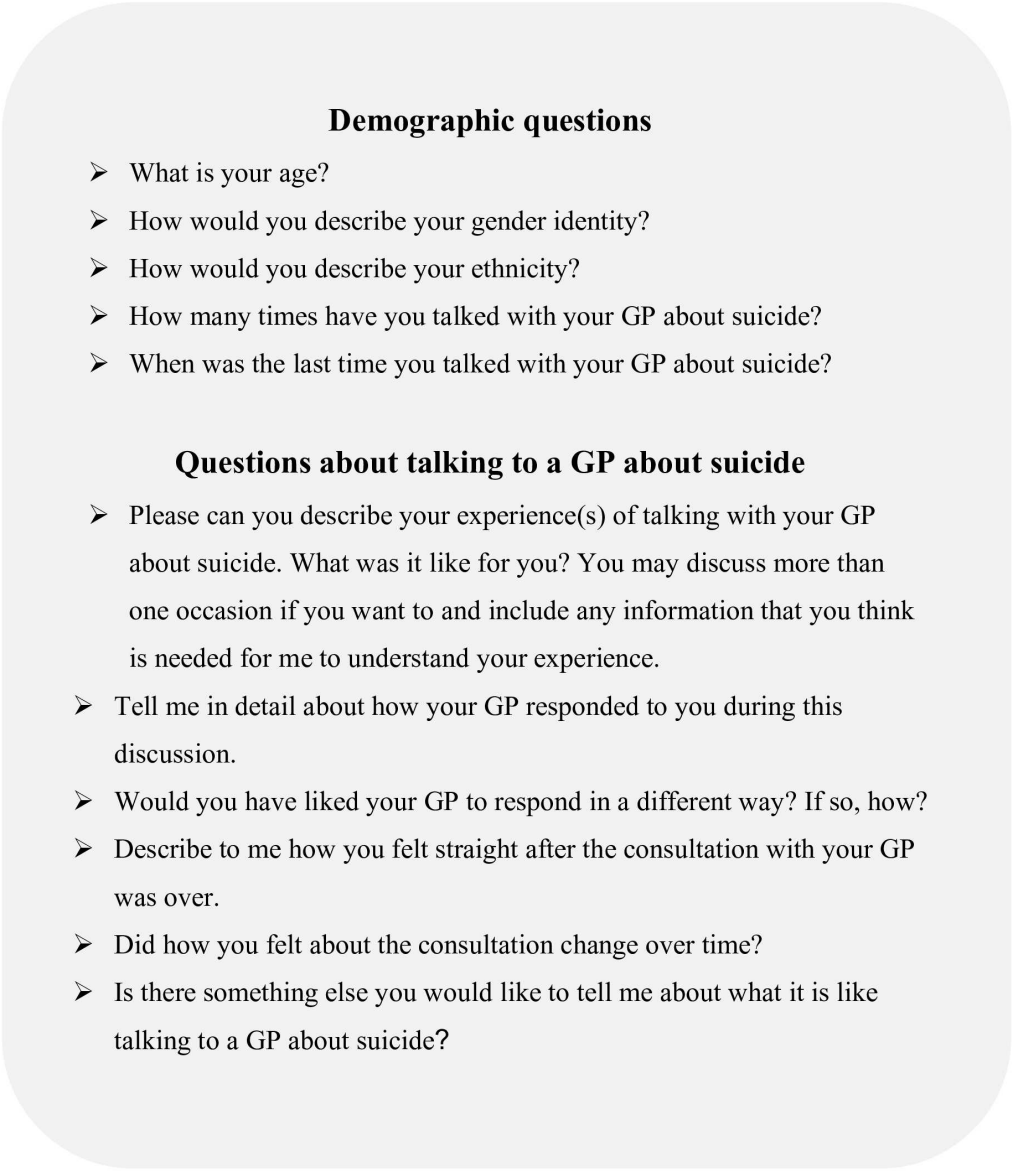
The survey questions developed in collaboration with lived experience volunteers.

A qualitive survey was suggested by the researcher following initial conversations with the steering group. The key points of discussion raised by the volunteers during these conversations were:

1. The value of offering participants the opportunity to share their experiences from the comfort of their own home, or other chosen safe space, and to leave their responses anonymously. Stigma, shame, and embarrassment were highlighted as barriers to people coming forward to talk about their experiences.
2. To safeguard against potential re-traumatisation, signposting to support services and being clear that taking part in the research was voluntary, were identified as important. Participants were able to contact the researcher and could leave a contact email for the researcher to get in touch with them should they have any concerns or follow up questions. Many of these points were are also covered by the University research ethics review and approval processes.
3. In terms of survey design, volunteers emphasised that participants need to be able to move backwards/forwards through the survey and miss out questions if they want to. This would give as much control as possible to the participant in completing their responses. Questions were developed by the researcher and amended following further discussion with the volunteers. **Figure 2** presents the final set of qualitative survey questions.

The survey was created using Qualtrics software and disseminated in 2020 during COVID-19 restrictions using social media. Participants who were over 18 years of age and had relevant experience of talking to a GP about suicide were sought. A detailed description of the aim of the study and how it was being conducted, who the research team were and how to contact them was included in the Participant Information Sheet provided to the participants. The participants were not able to access the survey questions until they had accessed this information and provided informed consent. Questions were designed to allow participants to respond freely, without prompts or examples, to avoid influencing responses.

### 2.4 Setting, participants and recruitment

Seventy-two people from the United Kingdom accessed the survey and forty-one provided usable responses. Incomplete surveys were included in analysis. Only survey responses that did not answer any of the questions about talking to a GP about suicide were excluded (n = 31).

Respondents were aged between 19 and 67 years old. Thirty-nine participants identified as White British, one Black British and one British Jamaican. Twenty-nine were female, nine male, two non-binary, and one did not disclose their gender. The average number of times a respondent had spoken to a GP about suicide was nine (range 1-60). The average time since respondents had spoken to a GP about suicide was 30 months (approximately 2.5 years) and ranged between 231 (approximately 19 years) and less than one month.

## 3 Analysis

Informed by Braun and Clarke, this study sought to establish credibility through reflexivity, transparent documentation of the iterative process, grounding in data, and careful, inductive theme development, rather than through standardised metrics or statistical validation (33, 34). Data were analysed using reflexive thematic analysis and focused on identifying and reporting patterns of meaning. This involved the following steps: (1) data familiarisation and writing familiarisation notes; (2) coding the data in a systematic way; (3) generating initial themes; (4) developing and reviewing themes, generating thematic “maps” of analysis; (5) refining, defining and naming themes, in a way that captures the overall story the analysis tells; and (6) producing the write up, using participant quotes, and relating the analysis back to the research aims (35, 36). All of this was underpinned by a critical realist approach which provides “access to situated, interpreted realities”, interpreted by both the participant during data collection and researchers during analysis (33). The initial coding was conducted by SF using NVivo. The aim was to conduct an inductive analysis grounded in the data and informed by discussions with volunteers with lived experience of suicidality across two reflective workshops. The analysis was further informed by critical suicidology principles, which foreground participants’ lived experiences, consider the social and structural contexts shaping suicidality, and emphasise reflexivity in interpreting meaning.

### 3.1 Positionality

SF is a White British woman in her mid-thirties from a working-class background. She has spent around a decade studying and working in higher education, with additional experience in the third sector, particularly in roles focused on community engagement and lived experience involvement. SF brings personal lived experience of suicidality and of seeking support through various systems over a number of years. This background informs her research interests in suicide prevention, primary care, and the broader socio-political contexts that shape help-seeking experiences. The first author’s dual perspective as both a researcher and someone with lived experience has shaped the design, conduct, and interpretation of this study. While this positioning brings valuable insight into the complexity of service user experiences, it also requires ongoing reflexivity to acknowledge how the author’s own experiences, assumptions, and values may influence the research process. In particular, SF recognises the importance of creating space for a range of participant narratives, including those that may differ from her own, and has engaged in regular dialogue with others with lived experience to challenge and enrich the study’s interpretation.

### 3.2 Workshop one

Volunteers were provided with excerpts from the survey responses along with the associated information that would be available to the research team (participant age, gender, ethnicity, how many times they had spoken to a GP about suicide, and an approximately how long ago this was). These were printed onto A3 paper to allow notes to be written alongside the excerpts. A “round table” discussion took place with SF and volunteers reading and reflecting on the excerpts. The following three questions were used to encourage discussion:

1. What are your first impressions after reading this excerpt?
2. What do you think are the key elements in this excerpt?
3. How would you summarise this excerpt in a word or short sentence?

The volunteers also shared their personal experiences of talking to GPs about suicide and discussed how these experiences inform their thoughts about the excerpts. Volunteers’ experiences contextualise the excerpts, supporting a more holistic and socially situated interpretation. The method respects and values lived experience as a legitimate source of knowledge. This can be empowering for volunteers and ethically significant, particularly in research areas like suicide, where participants and contributors are often pathologised or excluded from interpretive authority.

### 3.3 Workshop two

SF generated the initial themes prior to the workshop. During the workshop, SF provided an overview of the themes and some corresponding quotes and described how they had arrived at these themes. Volunteers provided feedback on the themes, reflected on how compared with their own experiences, reflected on the quotes.

Involving volunteers in reviewing the researcher-generated themes allows for experiential validation. When volunteers reflect on how the themes align (or not) with their own experiences, it enhances the credibility and relevance of the findings by ensuring they resonate with those most affected by the research topic. By opening up the analytical process to scrutiny, the researcher (SF) modelled reflexivity and invited constructive critique, respectful of the influence of their own positionality. Volunteer feedback helped to highlight assumptions, oversights, or blind spots in the thematic analysis, supporting a more nuanced and transparent interpretation.

## 4 Results

### 4.1 Themes

Three overarching themes were identified: 1) *Challenges disclosing suicidal thoughts and feelings*; 2) *Challenges for the GP*; 3) *Creating a safe* sp*ace* (**Table 1**). Each theme and corresponding subthemes are described below, supported by illustrative quotations tagged with a participant number to maintain anonymity.

**Table 1:** Overview of themes.

Theme	Name	Sub-themes
1	Challenges disclosing suicidal thoughts and feelings: “*I wish she would just say suicide*”	The challenge of disclosure Fear of not having control
2	GP limitations: “*I felt my medical needs were met, but not necessarily my mental health needs*.”	GPs’ limited options Asking in the right way
3	Creating a safe space: “*He made it normal, not embarrassing or weird.*”	Supported in hopelessness Creating conditions for honesty

### 4.2 Challenges disclosing suicidal thoughts and feelings: “*I wish she would just say suicide*”

The first theme describes a range of challenges experienced by participants in relation to disclosing suicidal thoughts and feelings to GPs. These challenges seem to be connected to the stigma associated with suicide and this theme explores how this impacts the experience of “disclosing” suicidality.

#### 4.2.1 The challenge of disclosure

Many participants discussed the challenges they faced when talking to a GP about suicide. Participants highlighted that they found it very difficult to talk to a GP about suicide when actively experiencing suicidal thoughts and feelings: *“I found it really hard to talk to them, they seemed to blame me and my lifestyle on how I was feeling” (P 20 F WB_2).* Some participants found it “*hard to put into words” (P 20 F WB)* how they felt at the time.

One participant said: *“It was the first time I’d talked to anyone in person about my feelings around suicide and self-harm so there was a huge wave of emotion.” (P 22 NB W).* Participant 22 indicated that this is the first time they had talked to anyone about feeling suicidal, not just a GP, and conveyed feelings of vulnerability associated with this experience. Disclosure of suicidal thoughts and feelings can be so distressing that it can cause physical reactions in participants, as one participant described: *“It was stressful, I broke down crying which triggered a nosebleed” (P 22 NB WB).*

One respondent said that their suicidal thoughts and feelings are intertwined with feelings of shame (*“I often feel shame for feeling it.” P 30 DNDG WB*). Another participant describes that disclosure of suicidal thoughts “*can feel like displaying a weakness, or letting someone into something very private” (P 28 F WB)*, which indicated the deeply personal nature of suicidal thoughts and feelings. This quote also highlights that the thought of disclosure, not just the act, can evoke powerful feelings of shame. This participant talks about disclosing suicidal thoughts to their GP as “*admitting to”* having those thoughts:

*“[I] found it very hard to broach the subject. I found saying ‘I feel low’ wasn’t too difficult, but admitting to suicidal thoughts was much harder.” (P 27 F WB_2)*.

The choice of language is suggestive of a confession as if to a crime or something socially distasteful, and indicative of an internalised sense of shame surrounding their experience of having suicidal thoughts.

The factors and life events that contribute to suicidal ideation are complex and nuanced, this was touched upon by a participant who states *“having lived through life shattering complex trauma. It’s hard to discuss” (P 28 F WB).* Disclosure of these life experiences and the current suicidal thoughts and feelings was challenging, and respondents described the strength they needed to see it through*: “I feel like discussing it takes such a strong willpower and isn’t easy.” (P 21 F WB).*

The expectation that GPs would initiate dialogue around suicide was common among participants, who frequently described “waiting to be asked” rather than feeling able to initiate the conversation themselves:

*“It wasn’t so much telling the GP that I was engaged with these thoughts and behaviours, more waiting to be asked after we’d discussed depression.” (P 26 F WB)*.

By GPs inviting the conversation about suicide, they signalled that it was acceptable and safe to do so for the person seeking support. When GPs demonstrated discomfort in discussing suicide, participants found it even more difficult to share their experiences and engage openly, some described their conversations as *“awkward”*.

*“She usually asks about suicidal thoughts during our appointments, but does not like to use the word suicide, or any other word surrounding it. This can make it a bit awkward. If I tell her, for example, that I’ve had some suicidal thoughts, at the next appointment, she won’t say, ‘are you still having suicidal thoughts?’ She will say ‘so, um, about what you were saying regarding those thoughts, um about those pills, how are you feeling about stuff like that?’ (P 28 F WB_2)*.

The same participant acknowledged the support that they receive from the GP but also talked about the impact it has on them when the GP finds it difficult to say “suicide”. This perceived reluctance on the part of the GP contributed to a sense that experiencing suicidal thoughts and feelings was something shameful, even within what should have been the safety of a medical appointment:

*“While I am very appreciative of her support, dancing around the word suicide like that is uncomfortable and makes me feel that it’s somehow dark and shady - something to be ashamed of and not* sp*oken about. I wish she would just say suicide.”* (P 28 F WB_2).

The participant was clear that they would have preferred the GP to be more direct when talking about suicide, and in doing so to make the subject less “dark and shady”.

#### 4.2.2 Fear of not having control

Not having control over what happens once the information was “out” was a concern for many participants who were anxious that the GP would contact other services or family members without their consent. There was a sense of a risk with disclosing suicidal thoughts to the GP, adding to the concerns about vulnerability associated with these disclosures.

*“It also feels like losing control to some extent - because once the information is out, the GP could call the crisis team/CMHT/a family member, and you can’t always control that, or decide who gets to know.” (P 28 F WB)*.

This participant talked about not being able to “*decide who gets to know”* that they are feeling suicidal, which in this situation seemed to conflict somewhat with them feeling the need to bring the GP into what they are experiencing and to seek support. There may be many reasons why participants do not wish to share this information with people outside of their consultation. However, participants in this study mostly talked about fear of the consequences of the GP sharing the information they disclose about their suicidal thoughts.

*“I was still paranoid as soon as I left that she would ring an ambulance for me or tell my parents. Was really concerned about confidentiality” (P 21 F WB)*.

A common worry for participants was the fear that they would be detained under the Mental Health Act: *“I was scared that I would end up being sectioned and sent to hospital. I mainly lied saying I wasn’t actively suicidal.” (P 21 F WB).* There was a sense that participants felt the GP would make the decision without talking with the participant about their decision-making process, that the participant would be “done to” rather than worked with; *“You feel like if you mention suicide, you will be put in a straitjacket and whisked away.” (P 19 F WB).*

One participant connected being detained under the Mental Health Act with losing liberty; *“When you’re distressed the last thing you need is to have your rights taken.*” (P 39 M BJ), suggesting this would not be beneficial for a person experiencing the distress associated with suicidal thoughts and feelings. Such worries about being detained under the Mental Health Act would exacerbate worries about the potential outcomes associated with disclosure of suicidal ideation, and the sense of social shame this may bring.

Fear of the consequences of disclosure led some participants to hide the full extent of their suicidal thoughts and feelings when talking to GPs.

*“I still refuse to say if I am actively suicidal and always play it down a lot as I am scared they will tell my parents or section me. I can never fully be honest. Feel like it never really helps” (P 21 F WB)*.

One participant highlighted that they feared disclosure would result in their children being taken into care: *“It’s particularly scary if [you’ve] got a child [because you’re] convinced they’re going to take the kid” (P 46 F WB)*. This put a barrier between the participant and the support they are seeking.

Participants’ fears of the consequences of disclosure were based on experience, as one described:

*“The locum GP rang an ambulance and acted as if I’d made an attempt on my life right there and then in front of him. I was devastated [ … ] As soon as I mentioned suicide to the locum, he instantly blew up and when he asked for a reason to stay alive, I couldn’t give him one because I was having an anxiety attack. It was horrifying. [ … ] I just wanted to end everything because his reaction made me so ashamed. Because I was 17 at the time, he rang my parents who I hadn’t told about my mental health. I felt humiliated.” (P 19 F WB)*.

While the GP may have believed they were doing the right thing to keep the participant safe, their reaction to a young person’s disclosure of suicidal thoughts and feelings resulted in the participants feeling *“humiliated” (P 19 F WB).* Other participants shared their feelings after a consultation they perceived negatively; “*left me feeling very hopeless” (P 20 F WB_2)*; *“distressed. Worthless. Weak. Hopeless. Lost.” (P 27 F WB_2)*. Control was taken away from them at a time when they were particularly vulnerable and looking for support:

*“People tell you to Reach out and get help but when you feel like you’ve been turned down it just makes everything seem pointless.” (P 20 F WB_2).*

Participants described significant challenges in disclosing suicidal thoughts, feelings, and behaviours within primary care. Shame associated with suicidality remained a persistent barrier, contributing to reluctance to seek help. Many participants expressed an expectation that general practitioners (GPs) should initiate conversations about suicide; however, where GPs appeared uncomfortable with the topic, this was perceived as a further obstacle to disclosure. Concerns were also raised about the potential consequences of disclosure, particularly that disclosure could lead GPs to make decisions about a participants care without including them, for example informing family members, initiating compulsory admission under the Mental Health Act, or actions that could result in the loss of child custody.

### 4.3 General practitioner limitations: *“I felt my medical needs were met, but not necessarily my mental health needs.”*

This theme examines the limitations that GPs experience during consultations about suicidal thoughts and feelings as viewed from the perspective of the participant.

#### 4.3.1 General practitioners’ limited options

There was recognition of variation between GPs’ knowledge of suicidal thoughts, feelings, and behaviours, which some participants associated with variation in resources available to the practice; *“in general practice there are limited resources and knowledge that varies wildly from doctor to doctor.” (P 30 F (DND) WB).* Others expressed feeling overlooked but did not attribute this to a lack of resources, rather that they felt disregarded by the GP: *“I understand they were trying their best due to limited resources but I feel I was overlooked massively” (P 20 F WB_3).*

In this climate of limited resources, participants identified a broader lack of training among medical professionals in assessing suicide risk, extending beyond GPs; *“Medical professionals are not trained in or comfortable with suicide assessment.” (P 43 M WB).* Mental health was often viewed as multifaceted and beyond the scope of a GP; *“GPs do wonderful things but Mental Health is so complex they cannot help.” (P 39 M BJ).* Another participant described the consultation as leaving their mental health needs unmet; *“I felt my medical needs were met, but not necessarily my mental health needs.”* This paucity in specialised knowledge can surface during consultations at crucial moments, such as in the GP’s response to someone disclosing suicidal thoughts and feelings: *“I felt like the GP didn’t really know what to say or how to react. He referred me on to some mental health service with a long wait time.” (P 22 NB WB).* One participant discussed their frustration at knowing why they were experiencing suicidal thoughts and feelings but not getting the support they needed: *“I need a way out of the domestic violence from my female partner at the time. This is when I got the ‘man up’ vibe. I said on one occasion ‘I’ll do it again if I don’t get out of the home’” (P 39 M BJ).* This participant clearly identified the main source of their distress and expressed disappointment that the GP’s response did not address it. Instead, they appeared to focus on the *symptom*, the suicidal thoughts and feelings, by suggesting medication, rather than engaging with the underlying cause. The participant noted experiencing a *“man up vibe”* affirming the social shame attributed to stereotyping around domestic violence and abuse; *“I feel GPs only wanted to give meds to suppress my emotions. Whilst people want to help, they don’t act on what I see as the main source.” (P 39 M BJ).* This approach reflected a medicalised understanding of suicidality, wherein emotional distress is framed as a clinical issue to be managed rather than as a response to complex social and environmental factors. It highlighted the limitations of a medical model in situations where broader, context-sensitive support may be more appropriate. The participant’s plea to *“Guide me to the people who would help to protect me” (P 39 M BJ).* underscores the critical need for supportive, relational care that goes beyond clinical assessment, highlighting a desire for trusted connections within a system often experienced as fragmented and dehumanising.

Central to this sub-theme was that participants identified this lack of connection as a limitation, and often commented on how the GP could have responded in a way that was more considerate of their situated experience:

*“He could have signposted me to resources [ … ] There must be local services that aren’t NHS that people could be told to e.g. put in their mobile for emergencies, when you can’t see a way out of complete despair.” (P 37 F WB).*

In this sub-theme participants felt that the GP has not understood their situation and so have not made appropriate decisions. In some situations, this contributed to increased hopelessness, and a perceived lack of appropriate support. Participants acknowledged the pressures of limited resources but also identified GPs’ limited knowledge and understanding of suicidality as a key challenge. They expressed a desire for GPs to be better connected with other resources in order to expand the range of support options available to those seeking help.

#### 4.3.2 Asking in the right way

One participant highlighted they perceived the response to disclosure can vary greatly between GPs: *“Different GP’s and professionals react in a different way.” (P 31 F WB).* Despite available guidelines to help GPs find the appropriate course of action, it appeared there were no standard responses. This presents a considerable barrier for people seeking support who must “take a risk” each time they disclose because they do not know how the GP will respond to them.

Some participants negatively perceived their interactions with their GP; “*very condescending he didn’t believe it was possible for a 15 year old to be suicidal.” (P 22 F WB_2)*, and *“the GP seemed dismissive and simply calculating a risk” (P 22 F WB).* Perceiving the experience as negative and unhelpful meant that some participants stopped seeking support from their GP: *“I would never consider seeing the GP any more as it is like banging your head against a brick wall.” (P 67 F WB).*

Participants characterised the questions GPs had asked them during their consultation as *“basic”* and stated *“ultimately, GPs don’t ask enough or in the right way.” (P 31 F WB).* Similarly, the way GPs spoke to participants had an influence on how they interpret the experience of talking with a GP about suicide; using medicalised language was viewed negatively *“It was very sterile and based on medical terms” (P 45 M WB)* and *“I was* sp*oken to more like a case rather than a person” (P 21 F WB_3).* Both quotes illustrate a desire for a more empathetic and understanding approach from the GP to avoid individuals leaving consultations with feelings of regret: *“[I felt] Heartbroken and let down. Felt like in a way I should have kept it all to myself.” (P 20 F WB)*,

In summary, experiences of disclosure varied considerably, with no clear evidence of a standardised response despite existing guidance. Unpredictability in responses contributes to worry about disclosure and in some cases leading to disengagement from GP services entirely. The use of medicalised language during consultations was experienced as dehumanising and, for some, caused regret about having disclosed suicidal distress. While participants recognised the pressures facing primary care, they identified substantial gaps in suicide prevention training and a lack of integration with non-clinical support pathways.

### 4.4 Creating a safe space: *“He made it normal, not embarrassing or weird.”*

Theme three illustrates some of the conditions that lead to participants describing positive experiences of disclosing their experiences of suicidality with their GP.

#### 4.4.1 Supported in hopelessness

Participants indicated that whilst GPs are limited in the solutions they can offer someone experiencing suicidal thoughts and feelings, their efforts were still appreciated: *“although he is very supportive it is very hard for him to provide a solution and he can only talk me through what can be done” (P 27 F WB).* In line with this, one participant described themselves as feeling: *“Hopeless but supported in that hopelessness.” (P 28 F WB).*

The role of the GP as an advocate was highlighted by one participant, depicting them as repeatedly trying to help them to access support by pursuing referrals with mental health services, which remain unsuccessful*; “My GP has done absolutely everything possible to try and advocate for me, I desperately need help to deal with the trauma.” (P28 F WB).* This participant acknowledged that the GP was doing their best but are unable to resolve everything for them. Unlike negative experiences (theme two), the situation described here alludes to a therapeutic alliance in which the GP used their power within the healthcare system to advocate for them.

Consistent contact with a GP over time was also highlighted as beneficial for building trust and for disclosures: *“My GP is very nice and supportive. We have very regular appointments and have for over a year” (P 28 F WB_2)*; *“He always asks me about mood on every visit. He was really caring.*” (P *50 F WB).*

Allowing the two parties to become familiar with each other can ease some of the concerns about confidentiality (Theme one); *“When you have a relationship built on mutual respect and honesty this is pretty life changing for someone in my situation I think, especially when mental health services are so dehumanising and treat you like a piece of dirt without fail.” (P 28 F WB).* This familiarity can help demystify roles, reduce perceived power imbalances, and support a sense of psychological safety. As a result, individuals may feel more confident that their disclosure will be handled sensitively and ethically, addressing some of the anxieties or reservations expressed earlier in relation to “who gets to know”. This familiarity also provided a certain amount of predictability in the relationship:

*“I have found its much more unpleasant having these conversations with an unknown GP, as you have no idea how they may respond. Having a long term relationship with one GP has made it much easier for me, as I know what she will say, and do.” (P 28 F WB).*

This predictability supports feelings of safety for participants creating a place where they can talk about the suicidal thoughts and feelings they are experiencing. By having the time to develop some familiarity with the GP across appointments, participants feel more confident in how the GP will respond to them talking about suicide during their consultations. This predictability supports feelings of safety for participants, creating a place where they can talk about the suicidal thoughts and feelings they are experiencing: *“We have very real discussions about the hopelessness of my situation and the pain that I am in.” (P 28 F WB)*.

#### 4.4.2 Creating conditions for honesty

Often participants had sought support from more than one GP and had experienced both positive and challenging consultations. Drawing on those comparisons led some participants them to describe themselves as “*lucky”:*

*“I feel more grateful and lucky to have that kind of support. I despise any conversations I’ve had in the past where GPs have demanded you go to A&E or called the police/ambulance. Or implied that’s your choice so go for it if you want it.” (P 28 F WB)*.

This account highlighted that participants appreciated supportive, non-coercive responses in contrast to previous encounters with healthcare professionals, where crisis interventions such as A&E referrals or police involvement were experienced as disempowering and distressingly punitive rather than caring. Such favourable experiences seem to be underpinned by the participant feeling they can be honest with their GP about how they are feeling: *“It is enormously helpful to have support through this and to be able to be honest.” (P 28 F WB)*. The GP facilitated this by taking a non-judgemental and open approach. This participant’s reflection highlighted the critical importance of relational care in primary settings.

*“I am indescribably lucky to have a GP like this who can sit with the reality of my situation and life, and the physical and emotional pain I am in, and talk honestly to me about it without jumping to we must section you.” (P 28 F WB)*.

The GP’s ability to hold space for emotional and physical pain may enable more open, constructive dialogue around suicidality and risk. P 28 F WBs account highlighted the participant’s appreciation for a GP who responds with empathy and honesty, rather than resorting to coercive measures, illustrating the significance of relational trust and non-pathologising approaches in mental health care.

The behaviour of the GP directly influences the way the person feels when talking about suicide. There were some indications of the features of the consultation that can have a positive influence on the interaction between the two parties: *“Direct questions and* sp*ace to talk (not jumping in to fill a pause), really helped me to be honest about how bad things were.” (P 37 F WB).* GPs guiding the discussion but not dominating it helped to create a sense of safety, allowing for honesty, thereby facilitating disclosures of suicidal thoughts and feelings; “*He made me feel at ease during the consultation and I knew I could go back if my thoughts turned down a dark path again.” (P 27 F WB).*

Participants identified listening as a key skill exhibited by GPs stating, *“My current GP is a lot more inclined to sit down and listen to my concerns.” (P 22 F WB_2).* Participants were also aware of body language, and general behaviours that indicated active listening:

*“She listens, without hammering away at her keyboard like other GPs, which is nice. If she needs to keep notes, she will say she’s just going to note a few things down so she can remember it all, and she writes it on a bit of paper - typing it up after I leave” (P 28 F WB_2).*

Participants valued GPs who created space for open dialogue, interpreting this as a signal of safety and acceptance. One participant shared, *“The GP let me* sp*eak, and encouraged me to tell him the full story” (P 28 F WB)* highlighting the importance of being invited to share without interruption. Such practice was viewed as indicative of the GP’s authentic engagement, creating a space where participants felt their disclosure was validated: *“He made it normal, not embarrassing or weird.” (P 50 F WB).* This approach was viewed as “life changing” for one participant:

*“Listened, empathised, was totally human and honest with me. When you have a relationship built on mutual respect and honesty this is pretty life changing for someone in my situation I think, especially when mental health services are so dehumanising.” (P 28 F WB)*.

The participants’ critical reflection emphasised the importance of relationships as a driver of transformation when grounded in mutual respect and honesty. The importance of this becomes especially apparent when contrasted with participants’ experiences of dehumanisation within wider mental health services, highlighting the critical role of relational care.

This theme summarises some of the key skills and approaches taken by GPs that participants found contributed to a positive experience when talking to their GP about suicide, including developing a therapeutic alliance over several appointments, promoting open and honest conversations about suicidal thoughts and feelings, and active listening. Participants expressed appreciation for GPs who offered support even when unable to provide direct solutions. Acts of advocacy, particularly when navigating other areas of the health system such as mental health services, were highly valued. Familiarity with a GP was also associated with greater predictability in interactions, contributing to a sense of safety when discussing suicide.

## 5 Discussion

This qualitative study used an online survey to explore individual experiences of talking to a GP about suicide to understand how they perceive these interactions. Understanding the perspectives of people experiencing suicidality may support improvements to risk assessment processes, while creating more opportunities for intervention. To this end, this study was developed with people with lived experience of suicidality.

The findings presented illustrate the range of experiences when talking to GPs about suicide and how the GP’s approach is key to the person feeling supported, particularly during difficult experiences of suicidal thoughts and feelings. In relation to this, the reflexive thematic analysis identified three main themes: 1) *Challenges experienced disclosing suicidal thoughts and feelings*; 2) *GP limitations*; and 3) *Creating a safe* sp*ace*.

Theme one highlighted challenges faced by people when disclosing suicidal thoughts and feelings to GPs, including the fear of what would happen after disclosure, and how the stigma associated with openly admitting and discussing experiences of suicidality impacts whether individuals disclose their experiences. Talking about suicide is very challenging. There have been several public campaigns aiming to dispel stigma and encourage openness and support (e.g., CALM, Samaritans, MIND). Yet many participants in the current study struggled with disclosing suicidal thoughts and feelings to their GP. They described feeling shame, displaying weakness, a fear of being detained under the Mental Health Act, and regretting making disclosures because of how their GP responded. This demonstrates the ongoing need for work to reduce the stigma around suicide, particularly in primary care which is often the last point of contact for many who die by suicide (6).

Participants expressed concern about “who gets to know” following disclosure of suicidal thoughts and feelings to a GP, citing fear of them breaching confidentiality and the consequences that might have (e.g., possible detainment under the Mental Health Act). Richards et al. (37) describes weighing up the fear of negative consequences against the hope for help as being involved in whether an individual decides to disclose suicidal thoughts and feelings. The current findings support this view as the participants demonstrated the “hope for help” by attending the GP consultation, despite fear of the consequences regarding “who gets to know” what they are experiencing. Following a negative experience, some reported that they would never again seek support from a GP; if considered in the context of Richards et al. (37), this adds another potential “negative consequence” to be considered that evidently tips the scale in favour of not disclosing fully for some.

Theme two identified the perceived challenges facing GPs during these consultations, in particular, the limited resources available to GPs when someone discloses suicidal thoughts and feelings, including their limited training and skills. This aligns with the lack of training and skill in primary care regarding suicide risk assessment and management highlighted in previous studies (12, 21). While this gap in training is a key challenge, literature indicates practice can be improved with intervention. A study which implemented a guidance manual for general practitioners showed significant improvements in perceptions and practices among those who used it, compared to those who did not (38).

Identifying factors such as personal and demographic information, could help GPs identify community assets to support individuals and provide additional resources for signposting (e.g., support groups and community organisations). Socio-cultural factors could be protective against suicide (14) and the literature shows people experiencing mental health challenges value having the choice of non-clinical alternatives and compared to public sector services, voluntary sector organisations were considered more responsive and adaptable to needs (39).

The way that questions about suicide are phrased has an impact on if and how a person discloses the nature of their suicidal thoughts and feelings. McCabe identified that most questions asked in primary care were framed in a way that communicated an expectation of no suicidal ideation (40). In addition, people were significantly more likely to say they were not suicidal when the question was negatively phrased and closed yes/no gateway questions were limiting, presenting barriers for the person seeking support (40). The current study highlighted that GPs asking questions about suicide directly led to participants perceiving the interaction with the GP more positively and created a sense of safety that facilitated disclosure.

Theme three gives specific examples of positive interactions highlighting key skills exhibited by GPs that enable a successful conversation about suicide in primary care. This theme focuses on how GPs were able to validate the participant’s feelings and use active listening to promote disclosure. GPs being able to create a safe space for the person to talk about their suicidal thoughts and feelings was considered necessary for honest and comfortable disclosure by participants. In a healthcare setting a disclosure of suicidal thoughts and feelings should always be taken seriously and met with empathy and understanding (20). Richards et al. (37) identified listening and caring as facilitators for discussions about suicide, and evidence supports taking a more narrative approach to assessment focussing on compassion and safety planning (20).

### 5.1 Strengths and limitations

A key strength of this study was the involvement of lived experience collaborators throughout the research process. Collaborators with personal experience of suicidality and seeking support in primary care played an important role in shaping the study design and delivery. Their input directly influenced the development of the qualitative survey, including the wording and framing of the questions, and the design of the participant information sheet to ensure accessibility and sensitivity. They also contributed to the analysis through their insights during reflective workshops, helping to ground findings in real-world experience and challenge assumptions and academic bias. This collaborative approach enriched the study by bringing depth and authenticity to the understanding of how people experience talking about suicide in primary care. Lived experience involvement is increasingly recognised as essential to the future of suicide prevention research, offering critical insight into the realities of suicidality and helping to shape more responsive, ethical, and effective approaches (41, 42).

Eliciting voices through qualitative research requires methodological approaches that actively challenge power dynamics to generate nuanced insights. Engagement with people with lived experience must extend beyond tokenistic or procedural inclusivity; it needs to involve a commitment to valuing diversity, recognising community knowledge and practices, and engaging with individuals as whole persons (43). Central to this approach is the establishment and maintenance of trustful relationships, which takes time and intentional effort. In the current study, this has involved sustained presence within communities spending time in local spaces, sharing coffee, and engaging in reciprocal storytelling (43). While such practices may appear informal, they are rarely adopted in conventional research and are crucial to establishing mutual respect and relational depth.

The use of an online survey opened up participation to anyone with access to the internet. This was beneficial due to the sensitive topic area and added an additional layer of anonymity as this method enabled people to share their experiences without face-to-face contact with the research team if they wish. This approach was endorsed by the community members with lived experience because this accessibility and anonymity, as well as the flexible navigation of the survey, supported feelings of control for the participant. Contrary to the assertions that qualitative surveys produce thin data, the current study elicited data detailing a broad range of experiences and stark emotional honesty that enabled a rich and nuanced understanding of participants perceptions of talking to a GP about suicide.

Although qualitative research is not intended to be generalisable, a limitation of the current study was that some features of the sample (i.e., the majority of respondents identifying as White British) suggest a need for additional research in other groups, as well as communities that may culturally define suicide in different ways. The current study is situated in a white, western understanding of suicide and, therefore, indirectly excluded the global majority.

## 6 Further research and clinical implications

There is a need for further qualitative research is needed to explore individual experiences in greater depth, particularly studies that meaningfully involve people with lived experience of suicidal thoughts and feelings throughout the research process. This can help ensure findings remain grounded in the realities of those most affected. There is also a need to engage with underrepresented groups, such as Black and minoritised communities, migrants, and people experiencing homelessness, to explore how cultural, structural, and social factors shape experiences of suicide, disclosure, and help-seeking. Understanding these diverse perspectives is essential to developing more inclusive, responsive, and equitable support systems.

The current study confirmed that using direct questions about suicide and a non-judgemental and understanding approach were key for participants to feel safe and able to disclose. Working with GPs and people with lived experience of disclosing suicidal thoughts, feelings, and behaviours, to co-create and deliver guidance and training for GPs to create these safe spaces and consider the social complexity of suicidality could improve participants experiences and lead to more successful intervention. As a part of this evolving approach to suicide prevention, we must recognise that GPs can be both healthcare practitioner and a person with lived experience of suicidality. This duality poses an opportunity for in-depth exploration of how this valuable understanding can be harnessed by practitioners through a “lived experience leadership” lens (44) to enhance practice and elevate experiential knowledge in approaches to suicide prevention.

## 7 Conclusion

The aim of this study was to explore experiences of talking to a GP about suicide to better understand how people perceive these interactions. The findings identify a range of factors affecting how people experience their discussions about suicide during GP consultations, including stigma, fear of the consequences of disclosing suicidality, the resources available to GPs generally, including training and active listening skills. This has implications for practice by indicating that people’s experiences are largely connected to how the GPs ask questions about suicide and create a safe space for disclosure of suicidal thoughts and feelings. Further in-depth qualitative work in partnership with people with lived experience, is needed to gain a greater understanding of experiences of seeking support for suicidal thoughts and feelings in primary care to support adaptations in practice.

## Data Availability

The datasets presented in this article are not readily available in accordance with the ethical consent provided by participants. Requests to access the datasets should be directed to f010904a@student.staffs.ac.uk.
